# An empirical study on impulse consumption intention of livestreaming e-commerce: The mediating effect of flow experience and the moderating effect of time pressure

**DOI:** 10.3389/fpsyg.2022.1019024

**Published:** 2023-01-16

**Authors:** Wei-wei Dong, Yong-qiang Wang, Jian Qin

**Affiliations:** School of Economics and Management, Shanghai Institute of Technology, Shanghai, China

**Keywords:** livestreaming information source, social presence, flow experience, time pressure, impulsive consumption intention

## Abstract

Based on the Stimulus-Organism-Response (S-O-R) model, this paper studies the impulsive consumption mechanism of consumers participating in livestreaming e-commerce from the perspectives of information source characteristics and social presence and examines the mediating effect of flow experience and the moderating effect of time pressure. Based on the consumption data of 268 live shoppers, multiple regression analysis and Bootstrap method were used to test the research hypothesis. The empirical results show that the credibility, professionalism, attractiveness, and interactivity of live information sources have significant positive impacts on consumers’ flow experience and impulsive consumption intention. Furthermore, coexistence, communication and emotional presence of social presence have a significant positive impact on consumers’ flow experience and impulsive consumption intention. Flow experience plays part of the mediating role in the process of the characteristics of livestreaming information sources and social presence affecting consumers’ impulsive consumption intention, while time pressure has a positive moderating effect in the relationship between livestreaming information source characteristics and coexistence presence and flow experience. The higher the time pressure, the stronger the promotion of live information source characteristics and coexistence presence on flow experience. This study enriches the research literature on the consumption driving mechanism of livestreaming e-commerce and offers practical enlightenment and reference to improve the effectiveness of livestreaming e-commerce anchors. It is also one of the first studies to apply the theory of flow and social presence to the impulsive consumption intention of livestreaming e-commerce.

## 1. Introduction

The wide application of social media and mobile apps expanded social networks *via* virtual interaction. Socializing and sharing is an intrinsic need of social media users who will gain a greater sense of belonging and participation than they will typically gain from simply shopping around from one website to another (Wang, 2021). E-commerce live stream is such a social way. It shares images, sounds and videos with the audience through digital technology and interacts in real time. Users rely on instant information to interact with anchors to create an immersive feeling (Chen and Lin, 2018). Unlike TV and YouTube streaming, live streaming is an interactive, engaging, user-centric, and synchronous environment that offers real-time human–computer-mediated interaction between viewer and streamer (e-vendor) as well as viewer-viewer (Kang et al., 2021). From the perspective of information dissemination, it is difficult for other technologies to achieve the rapidity of consumers’ access to information and the authenticity brought to consumers by live stream. With the rapid development of online live stream in recent years, consumers can choose more and more entertainment and shopping methods. Marketers are also looking for more attractive livestreaming e-commerce content to facilitate dissemination across digital channels and platforms (Liu et al., 2018). Data show that livestreaming e-commerce industry of China has made a huge leap since 2017, with the output value increasing by 400% year-on-year. By June 2021, the number of live webcast users in China had reached 638million, an increase of 75.39 million year-on-year, accounting for 63.1% of the total number of Internet users. Over 150,000 h of livestreaming are watched by consumers each day, and they buy more than 600,000 items on the live stream platform (China Internet Network Information Center, 2021).

The huge market potential and user base make many enterprises use live stream to attract users. Major e-commerce platforms such as Taobao, JD.com and Pinduoduo have also successively embedded livestreaming functions to quickly add new traffic ports. Watching the anchor showcase goods on the mobile platform and placing an order in the livestreaming room has become most consumers’ first choice, which has a profound impact on consumers’ shopping methods and shopping experience. At the same time, the livestreaming e-commerce usually designs interesting video content to demonstrate the use effect of the product, customers can also interact with other consumers in the process of participating in live stream, such as writing online comments, rewards, or shopping. This live stream ecosystem of real-time interactive social media can effectively influence customers’ impulsive consumption behavior (Shukla and Dubey, 2022). However, it is increasingly difficult for enterprises to retain consumers, due to the low conversion cost of livestreaming platform customers. Therefore, it is necessary to study the impulsive consumption intention of customers to participate in live e-commerce.

Some scholars have analyzed the influence mechanism of the information output subject on the information receiving subject from the perspective of the characteristics of the information source. From the perspective of the reliability and potency model of the information source in social psychology, Lu and Chen (2021) believes that information sources can influence audiences from three aspects: credibility, professionalism, and attractiveness. Later, on the basis of this theoretical framework, some scholars introduced the information source model into the live stream research to explore how the information source displays commodity information through multiple interactions in the live stream e-commerce, and then affects the dynamic mechanism of consumers’ decision-making in a scene-based manner (Park and Lin, 2020; Meng et al., 2021; Zhang M. et al., 2022). Some scholars have applied the concept of social presence to customer research and network interactions, and have actively discussed the research of social presence on human-computer interaction (Tsai et al., 2021), impulsive consumption intention (Zhang and Shi, 2022), perceived security (Lu et al., 2021), online transaction trust (Prahiawan et al., 2021), and hedonism (Lavuri et al., 2022) in network service improvement and community construction, virtual shopping center designs and online consumption decision-making. In the field of impulsive consumption research, Impulse consumption is described as a sudden, unplanned, compelling, and hedonic purchasing behavior that lacks deliberate consideration of all available information and alternatives (Lee and Chen, 2021). Research has shown that, compared to offline physical shopping, consumers are more likely to exhibit impulsive consumption behaviors when shopping online (Lo et al., 2022). Other scholars have studied impulsive consumption in the process of online shopping from the flow experience perspective (Hoffman and Novak, 2009; Chen and Lin, 2018; Li and Peng, 2021). For example, marketing strategy-related literature points out that the streaming experience can promote the consumption behavior of livestreaming users, because the selflessness and high interactive participation of consumers in the livestreaming process can make them fully involved, so that they can feel the strong excitement, fulfillment and happiness of the streaming experience, and make them unconsciously “plant grass” goods (Chen and Lin, 2018). In addition, studies have found that the main motivation of consumers’ online shopping is to save time and costs and for convenience (Sumarliah et al., 2022). The characteristics of livestreaming scenes can stimulate users’ streaming experience in a live stream, which affects their emotional dependence on live stream (Li and Peng, 2021), thereby driving them to engage in impulsive consumption behavior.

To sum up, the current research on livestreaming e-commerce mainly focuses on the analysis of factors related to live stream and is unable to give a more comprehensive and reasonable explanation for the decision-making process of consumers’ impulsive consumption intentions when watching a live stream. Moreover, most existing research on livestreaming delivery focuses on the data collection and analysis of short video platforms and pays less attention to platforms of livestreaming e-commerce. In addition, existing literature still pays insufficient attention to the mechanism of flow experience and time pressure on impulsive consumption in the context of live stream. Finally, from the perspective of livestreaming information source characteristics and social presence, what characteristics of the anchor can stimulate users’ feelings? How can social presence change user perceptions? By what mechanism does time pressure affect consumers’ flow experience and then make consumers form impulsive consumption intention? At present, the theoretical explanations and empirical analyses of these problems are relatively scarce. Therefore, drawing on the “S-O-R” model, this paper studies the mechanism of consumers’ impulsive consumption behavior in the livestreaming room from the perspective of the characteristics of livestreaming information sources and social presence, and examines the mediation mechanism of flow experience and the moderating effect of time pressure. The research results of this paper not only help to improve the competitiveness of anchors and enhance the effect of livestreaming marketing, but also enrich and expand the relevant research literature on the driving mechanism of customer impulsive consumption behavior in the process of live stream.

## 2. Theoretical background and literature review

### 2.1. Livestreaming information source

In the marketing communication model, the information source refers to the sender of information and the controller of information communication. During a live stream, the anchor actually acts as the sender and controller of information in livestreaming room. According to the theory of information source characteristics, information sources can affect their persuasion effect on the audience from three aspects: credibility, professionalism, and attractiveness (Lu and Chen, 2021). Existing literature points out that in view of the particularity of livestreaming information sources, in addition to the credibility, professionalism and attractiveness of traditional information source characteristic theory, interaction should also be included in the research context of livestreaming e-commerce (Wang et al., 2021). Specifically, credibility means the credibility of livestreaming input information. The more credible livestreaming information source is, the easier it is for users to internalize their psychology, which leads to consumers having a positive attitude toward information (Rungruangjit, 2022). The professionalism of the livestreaming information source refers to the relevant knowledge summarized by the anchor based on learning the cutting edge of fashion and a large number of use experience (Wang et al., 2021). The attraction of the livestreaming information source refers to the characteristics of livestreaming anchor, such as exquisite appearance, good figure and personality charm. The interactivity of the livestreaming information sources means that consumers can communicate and exchange information with anchors through public screens and timely feedback, allowing for emotional exchanges (Kang et al., 2021). This paper believes that these four characteristics of livestreaming information sources are independent of each other and work together on the inner feelings of consumers when watching a live stream, so that users can have a flow experience and form a sense of participation and interaction, thereby enhancing the strong attention and impulsive consumption intention of products in the livestreaming room.

### 2.2. Social presence

At first a stable attribute of media, the theory of social presence was defined as the significance of the relationship in interpersonal interaction in media (Coursaris and Sung, 2012). Later, with the development of distance education and social networks, many scholars questioned the media attribute of social presence, believing that users’ perception of the nature of media is more important than the attribute of media itself (Ming et al., 2021). Social telepresence is described as a kind of warm social interaction on the website, that is, the extent to which the media allows users to psychologically perceive the existence of others (Ying et al., 2022). Unlike traditional online shopping, live shopping has the characteristics of high real-time, high interaction and communication synchronization. In the context of live shopping, although consumers cannot truly communicate face-to-face, the characteristics of high timeliness and high interaction make consumers more empathetic and immersive. This strong sense of social presence can act on consumers’ psychology and behavior through a certain mechanism. This study intends to draw on Gunawardena’s theoretical division criteria of social presence in live stream marketing, and divide it into coexistence, communication, and emotional presence (Chen et al., 2021).

### 2.3. Impulsive consumption

Kollat and Willett (1969) first defined impulsive consumption as an unplanned consumption behavior, that is, consumers buy unplanned goods on impulse. Zafar et al. (2021) extended the definition of impulsive consumption, believing that it is a spontaneous, unreflective, immediate, and dynamic purchase tendency of consumers. Other literatures consider external stimulus factors and believe that impulsive consumption is triggered by external stimulus or generated by individual subjectivity, it is unplanned and accompanied by strong and lasting sudden consumption behavior (Zhang X. et al., 2022). So far, scholars have reached a certain consensus on the definition of the concept of impulsive consumption, believing that it is a behavior containing emotional and hedonic factors, which can better meet the emotional needs of consumers in the process of consumption, including all kinds of positive and negative conditions. Existing literature points out that compared to offline physical store shopping, consumers tend to show more impulsive consumption characteristics when shopping online. In livestreaming e-commerce, this behavior is mainly driven by an anchor’s product presentation or marketing stimulation: when consumers watch a live stream, they first generate an impulsive consumption intention (Zhang Z. et al., 2022), and then convert it into impulsive consumption behavior (Yu and Zheng, 2022).

### 2.4. Flow experience

The theory of flow was first proposed by Csikszentmihalyi (1975), which defined flow as a positive psychological state when an individual is fully engaged, focused, and involved in activities at a certain point in time. The generation of flow will be accompanied by a high sense of excitement and fulfillment. Existing studies have found that the flow experience can be regarded as an unconscious experience. In this state, users will ignore the existence of surrounding things, filter out unpleasant experiences, will not be disturbed by the outside world when performing tasks, and have a high degree of control over their own behavior. In the context of the Internet, Hyun et al. (2022) found that the flow experience has the characteristics of a sense of control, interest, concentration, spiritual enjoyment, internal skills, and personal challenges. A literature search found that at present, the theory of flow experience is mainly applied to the research of distance education, information technology, game experience and other fields, and its application in the research of livestreaming e-commerce is minimal. This paper believes that when consumers watch the live stream, the anchor relies on its own characteristics, adopts methods such as enthusiastic interaction and content display, so that users can participate in it, create an immersive feeling, and then make them indulge in it, concentrate on it, forget their troubles, have a flow experience, and generate an impulsive consumption intention under the guidance of the anchor and the rendering of the scene atmosphere.

### 2.5. Time pressure

Time pressure refers to individuals’ perception of time urgency caused by limited decision-making time in information processing or decision-making (Olschewski and Rieskamp, 2021). When conducting promotional activities, businesses often limit the time of activities, hoping to urge consumers to make purchase decisions under time pressure (Di Vaio et al., 2021). For example, the research of Sharma et al. (2020) found that time pressure can lead consumers to worry, anxiety, urgency, and other negative emotions, and change consumers’ preferences for deterministic and uncertain benefits. Chen et al. (2020) found that time limited promotions can stimulate consumers’ scarcity awareness of products or discounts, thereby improving consumers’ willingness to buy. Through their experiments, Peng et al. (2019) found that imposing a time limit during promotion is equivalent to sending a prompt signal to consumers, which can promote them to make consumption decisions as soon as possible. However, at present, there are very few studies on applying time pressure to e-commerce live stream scenarios.

### 2.6. Research model and hypotheses

**Figure 1:**
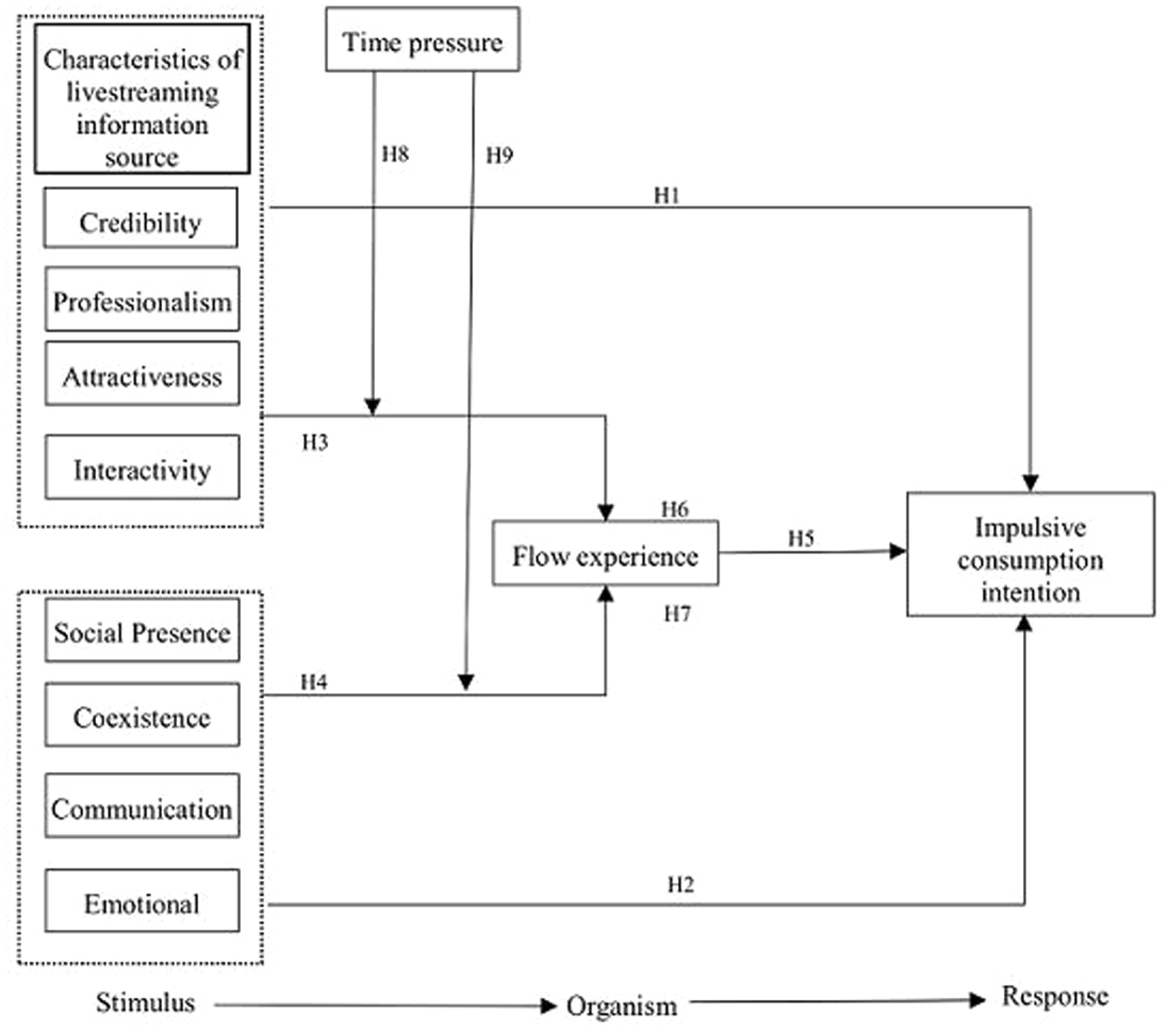
Research model.

The Stimulus-Organization-Response model (S-O-R model for short) is one of the important models in the field of psychology. This theoretical model studies the psychological and cognitive changes of individuals in the face of external environmental stimuli and the resulting individual behavior from the perspective of environmental psychology (Guo J. et al., 2021). According to the S-O-R model, consumers will be stimulated by a series of external factors such as livestreaming information sources in the process of watching a live stream, and then have a flow experience. On this basis, they will make individual responses, that is, produce impulsive consumption intention. Therefore, based on the S-O-R model and the theory of streaming experience, this paper takes the characteristics of live information sources as the antecedent variables, constructs the intermediate path based on streaming experience and the adjustment path of time pressure, and discusses the impact mechanism of the characteristics of live information sources on consumers’ impulsive consumption intention in livestreaming e-commerce. The research model of this paper is shown in Figure 1.

#### 2.6.1. The effects of livestreaming information source characteristics on impulsive consumption intention

Some studies believe that self-disclosure-high value online celebrity has a positive impact on impulsive consumption behavior. Professional knowledge-trusted online celebrity has a positive impact on impulsive consumption behavior (Chen et al., 2021). Therefore, in the process of watching the live stream, we will find that the anchor will make use of credibility and professionalism to make itself more credible and authoritative and attract consumers through their features such as appearance and talent and interact with consumers efficiently to make consumers have impulsive consumption intention.

Specifically, due to the complexity of products on the market, consumers are faced with a variety of product choices, and there is no time to have a detailed and careful understanding of each product, so they usually lack more comprehensive knowledge and information about a certain product. Consumers have come to trust and rely on UGC rather than the content companies generate on social media when making purchasing decisions. Companies not only promote the credibility of their products by sponsoring public figures and celebrities, but also create trust by fostering common attributes between ordinary consumers and independent content creators (Muda and Hamzah, 2021). Therefore, before purchasing a commodity, consumers may seek commodity information from experienced sources to judge whether the commodity meets their immediate needs.

And then, when the anchor recommends products in the livestreaming room, he usually has sufficient professional knowledge of such products. Consumers can get more product information from the anchor, to reduce the risk of consumption decision-making and increase consumption intention. Shen (2021) explores how fashion online celebrity, and its electronic word-of-mouth (eWOM) information can improve consumers’ participation in social media and draws the conclusion that the information professionalism of fashion micro-online celebrity will attract consumers to purchase intention to a certain extent.

In addition, the anchors in the live stream room are usually good-looking stars and online celebrity. These anchors are naturally attractive to consumers, which is becoming more and more attractive with the large number of social media users. Research by Dinh and Lee (2022) points out that the increasing use of social media makes the influence of social media influencers on consumers’ purchasing decisions increase, and consumers regard influencers as role models they try to imitate.

Finally, Lee and Eastin (2021) Research shows that a more flexible interactive framework in social media will help users form a closer relationship with brand marketing communication organizations and brand customers’ decision makers, thus increasing impulsive consumption intention. Content information in highly interactive (and less interactive) electronic screens has a positive impact on impulsive access and impulsive consumption through self-agency (Moes et al., 2022). The effective interaction between information sources and users in the live stream room may also promote users’ impulsive consumption intention.

To sum up, this paper puts forward the following assumptions:

*H1*: The characteristics of livestreaming information sources have a positive impact on impulsive consumption intention.

*H1a*: The credibility of livestreaming information sources has a positive impact on impulsive consumption intention.

*H1b*: The professionalism of livestreaming information sources has a positive impact on impulsive consumption intention.

*H1c*: The attraction of livestreaming information sources has a positive impact on impulsive consumption intention.

*H1d*: The interaction of livestreaming information sources has a positive impact on impulsive consumption intention.

#### 2.6.2. The effect of social presence on impulsive consumption intention

In livestreaming e-commerce marketing environment, businesses shape the situation through the e-commerce live stream platform, enhance the sharing and communication methods between enterprises and others, improve consumers’ sense of social presence when watching the live stream, and affect consumers’ purchase decisions and behaviors (Ming et al., 2021). Specifically, in the internet traditional e-commerce shopping experiment, Wang et al. (2021) found that social presence can effectively promote the generation of traditional e-commerce shopping behavior. In addition, social presence has also become a key factor affecting consumers’ impulsive consumption. Zafar et al. (2021) found that consumers’ awareness can be generated by telepresence when browsing websites, and this awareness can promote consumers’ impulsive consumption intention.

Firstly, in the internet traditional e-commerce shopping experiment, Wang et al. (2021) found that social presence can effectively promote the generation of traditional e-commerce shopping behavior.

Secondly, social presence has also become a key factor affecting consumers’ impulsive consumption. Zafar et al. (2021) found that consumers’ awareness can be generated by telepresence when browsing websites, and this awareness can promote consumers’ impulsive consumption intention.

Thirdly, compared with traditional marketing, the interpersonal relationship in the live stream marketing situation is more realistic, the communication is more real-time and interactive, and the emotions are more infectious and two-way interactive, which facilitates impulsive consumption intention (Zhang and Shi, 2022).

To sum up, this paper puts forward the following assumptions:

*H2*: Social presence has a positive impact on impulsive consumption intention.

*H2a*: Coexistence presence has a positive impact on impulsive consumption intention.

*H2b*: Communication telepresence has a positive impact on impulsive consumption intention.

*H2c*: Emotional presence has a positive impact on impulsive consumption intention.

#### 2.6.3. The effects of livestreaming information source characteristics on cardiac flow experience

Al-Emadi and Ben Yahia (2020) argues that celebrity endorsement of products or services can add value to products and make brands more famous, and their success through their own characteristics and exogenous efforts will have a positive impact on consumers’ psychology. The anchor of the live stream room will have the same psychological effect on consumers.

Firstly, the credibility of livestreaming information source refers to the audience’s trust in the anchor’s individual and the goods introduced during the anchors’ live stream. The more credible the information source is, the easier it is for individuals to psychologically internalize, which leads to the audience having a positive receiving attitude toward the information. At present, mobile Internet and digital technology have doubled the amount of information. Zhang S. et al. (2022) reveal that credibility can be enhanced through live interactivity (active control, two-way communication, synchronicity) and technical enablers (visibility, personalization), consequently affects continuance intention, in addition, live stream genres moderate the impact of different types of credibility on continuance intention. Consumers need to spend a lot of time and energy to screen information, compare and screen the goods they want to buy. First, during the live stream, if consumers have confidence in the products recommended by the anchor (quality, price, etc.), they will be more easily attracted by the anchor, resulting in a sense of pleasure. In other words, the higher the credibility of the anchor, the stronger the user’s sense of flow experience.

Secondly, during the live stream, the anchor will professionally explain and effectively evaluate the products, give consumption suggestions, and increase the excitement and interest of the audience when watching the live stream. Therefore, the stronger the professionalism of the anchor, the more professional information consumers have about the product. The less cost and energy they expend to search for the target product, the more conducive to improve consumers’ sense of experience, pleasure and focus on the product and livestreaming viewing. At a deeper level, people have the desire to understand unknown areas. Professional explanations will attract viewers to bring them into specific scenes, so that consumers can have a positive emotional experience (Akdevelioglu and Kara, 2020). Therefore, the more professional the anchor is, the easier it is to stimulate the audience’s flow experience.

Thirdly, consumers tend to have positive feelings for attractive anchors and transfer this positive attitude to commodities. Leite and Baptista (2021) suggests that online celebrity’s intimate self-disclosure to consumers will promote the attractiveness of its endorsement brands to consumers. The stronger the attraction of the anchor, the easier it is to establish a good interpersonal relationship with the consumers who watch the live stream, which is conducive to the audience’s love and identification with the anchor and their products. Other studies suggest that digital video storytelling is more attractive to consumers (Coker et al., 2021). In the virtual live stream scene with a good audio-visual effect and a symbolic anchor image, it is easier for the audience to get an immersive aesthetic experience, and it is also easier to produce a sense of engagement and pleasing pleasure, to produce a flow experience.

Finally, real-time interaction can also arouse the desire of consumers to participate, so that the audience can have a full range of sensory feelings, free from time and place constraints, and enhance the audience’s sense of control and substitution for live activities (Kim et al., 2021). Bao and Zhu (2022) believe that real-time interactivity is the premise factor of perceived value and customer satisfaction of live stream business platform, and then affects customers’ sticky willingness. Some scholars believe that interactive marketing stimulates the interaction between consumers and brands to increase consumers’ social value and generate impulsive consumption intention (Souki et al., 2022). Similarly, in the live stream, the stronger the interactive ability of the anchor, the higher the interactive frequency, the stronger the audience’s streaming experience, and the easier it is to produce consumption desire.

To sum up, this paper puts forward the following assumptions:

*H3*: The characteristics of live information sources have a positive impact on the flow experience.

*H3a*: The credibility of live information sources has a positive impact on the flow experience.

*H3b*: The professionalism of live information sources has a positive impact on the flow experience.

*H3c*: The attraction of live information sources has a positive impact on the flow experience.

*H3d*: The interaction of live information sources has a positive impact on the flow experience.

#### 2.6.4. The effect of social telepresence on flow experience

Existing literature shows that social presence in virtual situations will significantly affect the flow experience of network users (Wang et al., 2021). Firstly, Koponen and Rytsy (2020) found that B2C (business to customer) model can significantly enhance users’ sense of social presence by using virtual characters, promote their enhanced flow experience, to bring about a positive attitude toward the website. Other scholars have found that the strong interaction of virtual websites can promote social presence, which will further promote individuals to have a higher flow experience under the pleasure (Jiang et al., 2019).

In addition, compared with the above situations, the live stream marketing situation creates a better experience for consumers to perceive existence and interaction (Wongkitrungrueng et al., 2020), and participants can have a strong sense of existence. In the live stream scenario, consumers can realize synchronous interaction with other parties. Compared with asynchronous interaction, consumers will feel a stronger flow experience in the process of synchronous interaction (Cai and Wohn, 2019).

Finally, the stronger the sense of social presence, the more the emotions of all parties in the live stream room can affect each other, and the easier it is for consumers to obtain the flow experience (Tsai et al., 2021).

To sum up, this paper puts forward the following assumptions:

*H4*: Social presence has a positive impact on the flow experience.

*H4a*: Coexistence telepresence has a positive impact on the flow experience.

*H4b*: Communication telepresence has a positive impact on the flow experience.

*H4c*: Emotional presence has a positive impact on the flow experience.

#### 2.6.5. The effect of flow experience on impulsive consumption intention

Previous studies have shown that experience activities such as customer participation, customer learning and customer entertainment in shopping can make consumers produce a series of positive emotional reactions, and then stimulate consumers’ willingness to consume (Kühn and Petzer, 2018; Hsu, 2020). Compared to offline physical store shopping, online shopping can bring consumers more sense of control (time, place, etc.) and participation (interaction with anchors, comments, etc.). Compared to traditional online shopping, consumers can enjoy more freedom and control when entering the live stream shopping platform, which will also bring them a better consumption experience and make them have impulsive consumption intentions. Therefore, based on the above analysis, we put forward the following hypothesis:

*H5*: The intensity of flow experience has a positive impact on customers’ impulsive consumption intention.

The audience’s flow experience will have an impact on consumers’ shopping attitude: when users are immersed in the live stream environment, they will unconsciously want to participate in it, and produce impulsive consumption intention under the display and guidance of the anchor (Liu et al., 2022). The content generated by social media influencers is a powerful form of electronic word of mouth, which makes users feel more professional and credible, and then forms impulsive consumption intention (García-de-Frutos and Estrella-Ramón, 2021). During the live stream, the popularity and professionalism of the anchor can reduce the time cost for consumers to search for product information, allow users to watch the live stream more efficiently, and improve users’ shopping experience and pleasure.

In addition, the good appearance, physique, and personality charm of the anchor in the live stream room can attract more users, bring users visual or emotional aesthetic experience, wake up their sense of pleasure, and produce a flow experience (Wu et al., 2020). Frequent interaction between live stream rooms can also make consumers temporarily divorced from reality, immersed in the live stream environment, temporarily forget their troubles, and produce a flow experience. Some scholars have verified the positive effects of content quality (i.e., Visual aesthetic text information and timeliness) and system quality (i.e., Intuition and interactivity) on consumers’ flow experience (Cuevas et al., 2021).

To sum up, this paper puts forward the following assumptions:

*H6*: Flow experience plays a mediating effect in the influence of livestreaming information source characteristics on impulsive consumption intention.

*H6a*: Flow experience plays a mediating effect in the relationship between the credibility of live information sources and impulsive consumption intention.

*H6b*: Flow experience plays a mediating effect in the impact of live information source professionalism on impulsive consumption intention.

*H6d*: Flow experience plays a mediating effect in the influence of the interaction of live information sources on impulsive consumption intention.

*H6c*: Flow experience plays a mediating effect in the influence of the attraction of live information sources on impulsive consumption intention.

Animesh et al. (2011) verified that in the online virtual purchase situation, social presence positively affects the generation of online consumers’ flow experience, in turn affecting impulsive consumption intention. The relationship between flow experience and consumers’ impulsive consumption intention shows that the three dimensions of social presence, namely coexistence, communication, and emotion, affect the generation of flow experience (Guan et al., 2022). In the context of live e-commerce, the stronger the social presence, the more it can trigger consumers’ flow experience, and flow experience has a positive impact on impulsive consumption intention (Wang et al., 2021). Through research, Ming et al. (2021) have concluded that consumers’ coexistence perception, communication perception and emotional social existence will make consumers have flow experience and enhance their impulsive consumption intention.

Therefore, this paper proposes the following hypotheses:

*H7*: Flow experience plays a mediating effect in the influence of social presence on impulsive consumption intention.

*H7a*: Flow experience plays a mediating effect in the influence of coexistence presence on impulsive consumption intention.

*H7b*: Flow experience plays a mediating effect in the influence of communication telepresence on impulsive consumption intention.

*H7c*: Flow experience plays a mediating effect in the relationship between emotional telepresence and impulsive consumption intention.

#### 2.6.6. The moderating role of time pressure

Time pressure plays an important role in the formulation of consumer purchase behavior in the context of real-time interaction and rapid decision-making (Chowdhury et al., 2019). Haynes (2009) research shows that different time pressures will lead individuals having different degrees of information decision-making resources. Faced with miscellaneous information and information overload, consumers sometimes must spend a lot of time filtering information, comparing, and choosing the products they want to buy. Time pressure may cause consumers to have an “opportunity cost perception” in other words, consumers who do not buy goods within the specified time will “regret refusing to buy” (Tykocinski and Pittman, 2001). Time constraints create a sense of urgency that encourages consumers to buy (Inman and McAlister, 1994).

And then, when the anchor of the live stream room stimulates consumers to realize the value of goods and must make a purchase decision within the specified time, consumers will form psychological pressure (Peng et al., 2019). This means that the characteristics of anchors, including professionalism, credibility, attractiveness, and interactivity, are another potential cause of time pressure. In addition, customers may not have enough time to collect product evaluation information under time pressure, which will hinder information processing. The source of information mainly depends on the information source in the live stream room, which will stimulate the formation of consumers’ flow experience. In this way, consumers make the flow experience deviate through the influence of the information source in the live stream room (Guo L. et al., 2021). We believe that time pressure has a certain promotion mechanism between the characteristics of live information sources and flow experience, and based on this, we put forward the following hypotheses:

*H8*: Time pressure positively regulates the impact of live information source characteristics on cardiac flow experience.

*H8a*: Time pressure plays a positive moderating role between the credibility of live information sources and the flow experience.

*H8b*: Time pressure plays a positive moderating role between the professionalism of live information sources and the flow experience.

*H8c*: Time pressure plays a positive moderating role between the attraction of live information sources and the flow experience.

*H8d*: Time pressure plays a positive moderating role between the interaction of live information sources and the flow experience.

Time pressure is not only a cognitive awareness of not having enough time, but also a hurried and depressed emotional experience (Szollos, 2009), which can adjust the relationship between social network use behavior and perceived fatigue (Guo et al., 2020). Under strong time pressure, users will feel that they do not have enough time to watch the live stream. This urgent time perception will lead to changes in users’ sense of social presence, which will affect users’ flow experience when watching a live stream.

Shopping in the live stream room requires consumers to make purchase decisions in a short time, which may lead to time pressure because the available time is less than the time usually required to make such decisions (Peng et al., 2019). Consumers are likely to become anxious and stressed in this presence.

To sum up, this paper puts forward the following assumptions:

*H9*: Time pressure positively regulates the impact of social presence on flow experience.

*H9a*: Time pressure plays a positive moderating role between coexistence telepresence and flow experience.

*H9b*: Time pressure plays a positive moderating role between communication telepresence and flow experience.

*H9c*: Time pressure plays a positive moderating role between emotional presence and flow experience.

## 3. Research design

### 3.1. Sample and data collection

WeChat is the most widely used social media platform in China (with about 1.2 bn active users), and the Moments (a posting function of WeChat, like Instagram) is one of its most welcome functions. This study collected data on consumer groups who have experienced online livestreaming (Liu et al., 2021). Therefore, we choose the circle of friends of WeChat users with different age gradients to investigate whether users in different circles of friends have watched live shopping or placed orders in the live stream room. In February 2022, we published a questionnaire in a circle of friends and collected 334 questionnaires for 1 month. After excluding incomplete questionnaires, 268 valid questionnaires were finally determined, with an effective recovery rate of 80%, and the effective sample size was about 8 times that of the measurement items. The descriptive statistical characteristics of the sample are shown in Table 1, among them are the respondents who have experienced live shopping. Most of the time, they had participated in live shopping within 3 years, and the proportion who had done so over 3 years ago was 12.7%. Nearly half of the respondents’ monthly income was less than 3,000 yuan, and most consumers spend 50–500 yuan in the price range of the live stream room. In terms of gender, age, education and occupation, the sample of respondents had a high coverage rate of netizens, which ensures the quality of the questionnaire.

**Table 1:** Demographic statistics of survey samples (*n* = 268).

Sample characteristics	Classification criteria	Sample		Sample characteristics	Classification criteria	Sample	
		Number	Proportion/%			Number	Proportion/%
Gender	Male	96	35.8	Monthly income/yuan	Less than 3,000	129	48.2
	Female	172	64.2		3,000 ~ 5,999	44	16.4
Age	Under 20	7	2.6		6,000 ~ 9,000	44	16.4
	20 ~ 24 years old	139	51.9		More than 9,000	51	19.0
	25 ~ 29 years old	35	13.1				
	30 ~ 40 years old	45	16.8	Contact live shoping years/year	Less than 1 year	38	14.2
	Over 40	42	15.7		1 ~ 2 years	123	45.9
Education	Junior college or below	16	6.0		2 ~ 3 years	73	27.2
	Underguaduate	158	59.0		3 ~ 5 years	26	9.7
	Master	65	24.2		More than 5 years	8	3.0
	Doctor	29	10.8				
Occupation	Full-time student	140	52.2	Annual consumption price range/yuan	0 ~ 50 yuan	44	16.4
	Teachers or researchers	55	20.5		50 ~ 100 yuan	84	31.4
	Civil servant	10	3.7		100 ~ 500 yuan	119	44.4
	Enterprise staff	44	16.4		500 ~ 1,000 yuan	14	5.2
	Professional	8	3.1		Over 1,000 yuan	7	2.6
	Other	11	4.1				

### 3.2. Measures

The research variables in this paper were measured with a 5-level Likert scale and were refined and improved by reverse translation combined with team discussion. The characteristics of livestreaming information source adopts Ohanian’s scale (Ohanian, 1991). Considering the characteristics of live stream combined with the characteristics of information source, the questions were appropriately modified. Social telepresence adopted the scale devised by Gunawardena and Zittle (1997). The flow experience adopted the scale developed by Koufaris (2002), which contains 5 items in total. Time pressure measurement refers to the research of Lin and Chen (2013), with a total of 4 items. Impulsive consumption intention refers to the scale developed by Beatty and Ferrell (1998), which contains three items. The specific contents of the scale are shown in Table 2.

**Table 2:** Variables and items (*n* = 268).

α, CR, AVE	Measurement items	Factor loadings
Credibility *α* = 0.791 CR = 0.800 AVE = 0.571	Do you think the content of the live broadcast is credible	0.782
	Do you think the corresponding products recommended by the anchor are more reliable	0.736
	You trust the live broadcast platform / anchor you watch	0.749
Professionalism *α* = 0.729 CR = 0.752 AVE = 0.502	Do you think the live anchor you watch has professional knowledge	0.721
	Do you think the live anchor you watch is professional	0.713
	Do you think the live anchor you watched has rich experience in using the recommended products	0.692
Attractiveness *α* = 0.702 CR = 0.851 AVE = 0.656	The reason why you watch the live anchor is that the appearance of the anchor attracts you	0.811
	The reason why you pay attention to this anchor is that he (she) is very charming	0.805
	Do you think the live content of the live anchor you watch can arouse your interest	0.813
Interactivity *α* = 0.805 CR = 0.669 AVE = 0.503	Do you think the live anchor you watch has a good interactive relationship with you	0.705
	Do you think the live content of the live anchor you watch can enable you to participate effectively	0.713
Coexistence presence *α* = 0.790 CR = 0.755 AVE = 0.506	I feel that there is a cross time and space connection with others in the live broadcast room	0.714
	I feel online at the same time as others	0.723
	I feel as if I’m in the midst of a live broadcast	0.697
Communication presence *α* = 0.820 CR = 0.855 AVE = 0.597	I feel social in the studio	0.783
	I can continuously exchange views with others in the live broadcast room	0.790
	I can smoothly exchange views with others in the live room	0.780
	My questions can be answered by others in the live studio	0.736
Emotional presence *α* = 0.880 CR = 0.820 AVE = 0.603	Others in the live broadcast room bring me a warm feeling	0.781
	Others make me sensitive in the live broadcast room	0.803
	The happiness and anger of others in the live broadcast room affect my mood to a certain extent	0.745
Flow experience *α* = 0.849 CR = 0.833 AVE = 0.501	When watching the live broadcast of the anchor, I am highly focused (immersed)	0.644
	When watching the live broadcast, I sometimes ignore what is happening around me	0.760
	When watching the live broadcast of the anchor, sometimes I forget what I will do	0.721
	When watching the live broadcast of the anchor, I feel that everything is under control	0.686
	When I watch the live broadcast of the anchor, I feel very happy	0.720
Time pressure *α* = 0.790 CR = 0.800 AVE = 0.501	I have to act quickly for fear of missing the second kill during the live broadcast	0.678
	As the countdown to panic buying approaches, I feel very stressed	0.768
	I feel in a hurry when shopping live	0.688
	I do not have enough time to participate in live shopping	0.693
Impulse consumption intention *α* = 0.832 CR = 0.810 AVE = 0.587	After watching the live broadcast, even if the goods are not within the scope of my planned shopping, I still cannot help but want to buy the goods recommended by the anchor	0.745
	When watching the live broadcast, I experienced many impulses to consume goods	0.798
	When watching the live broadcast, I wanted to consume this product without careful consideration	0.755
Goodness of fit	*X*^2^ = 1041.556, *df* = 450, *X*^2^/*df* = 2.315^***^, TLI = 0.903, CFI = 0.941, RMSEA = 0.040	

*p* < 0.001, *p* < 0.01, *p* < 0.05.

### 3.3. Reliability and validity analysis

SPSS 26 was used to analyze the reliability of the questionnaire data (the results are shown in Table 2). The Cronbach’s Alpha of each variable is higher than 0.7. At the same time, the load factor of each variable is greater than 0.5, and the CR value is also greater than 0.7, indicating that each variable has good reliability. Secondly, Amos 26 was used to conduct confirmatory factor analysis on the model variables (results in Table 3). The fitting indicators of the seven-factor model (Chi-Square = 1041.556, *df* = 450, TLI = 0.903, CFI = 0.941, RMSEA = 0.040) were significantly better than those of other competitive models. The average refined variance (AVE) of each variable was greater than 0.5, and the square root of AVE was also greater than the absolute value of its corresponding correlation coefficient. This shows that each variable has good discrimination validity and aggregation validity. Finally, the relationship between the main variables is preliminarily analyzed through partial correlation analysis (as shown in Table 4).

**Table 3:** Results of the confirmatory factor analysis (*n* = 268).

Model	*X^2^*	*df*	*X^2^*/*df*	RMSEA	RMR	CFI	TLI
Ten factor model	1041.556	450	2.315	0.040	0.045	0.941	0.903
Nine factor model	1176.813	459	2.564	0.077	0.068	0.855	0.833
Eight factor model	1388.002	467	2.972	0.086	0.074	0.814	0.790
Seven factor model	1450.416	474	3.060	0.088	0.073	0.803	0.781
Six factor model	1515.239	480	3.157	0.090	0.071	0.791	0.770
Five factor model	1924.226	485	3.967	0.105	0.080	0.710	0.684
Four factor model	1976.825	489	4.043	0.107	0.081	0.700	0.676
Three factor model	2061.472	492	4.190	0.109	0.082	0.683	0.660
Two factor model	2184.541	494	4.422	0.113	0.084	0.659	0.635
Single factor model	2337.246	495	4.722	0.118	0.085	0.628	0.603

*p* < 0.001, *p* < 0.01, *p* < 0.05.

**Table 4:** Correlation matrix (*n* = 268).

	1	2	3	4	5	6	7	8	9	10
1. Credibility	**0.756**									
2. Professionalism	0.666^**^	**0.709**								
3. Attractiveness	0.417^**^	0.487^**^	**0.810**							
4. Interactivity	0.511^**^	0.518^**^	0.459^**^	**0.710**						
5. Coexistence	0.447^**^	0.501^**^	0.416^**^	0.670^**^	**0.711**					
6. Communication	0.439^**^	0.485^**^	0.390^**^	0.660^**^	0.750^**^	**0.773**				
7. Emotional	0.247^**^	0.317^**^	0.355^**^	0.391^**^	0.509^**^	0.570^**^	**0.777**			
8. Flow experience	0.360^**^	0.463^**^	0.480^**^	0.541^**^	0.584^**^	0.583^**^	0.521^**^	**0.708**		
9. Time pressure	0.455^**^	0.450^**^	0.432^**^	0.450^**^	0.467^**^	0.429^**^	0.532^**^	0.565^**^	**0.708**	
10. Impulse consumption	0.369^**^	0.383^**^	0.335^**^	0.275^**^	0.318^**^	0.338^**^	0.426^**^	0.520^**^	0.573^**^	**0.766**

*p* < 0.001, *p* < 0.01, *p* < 0.05; Diagonal cells in bold report the square root of AVE.

### 3.4. Tests of common method variance

Herman’s single factor method was used to test the possible common method deviation. After an exploratory factor analysis of all items of the research variables, the results showed that the variance contribution rate of the first principal component interpretation without rotation was 34.204%, indicating that the common method deviation did not have a serious impact on this study.

## 4. Hypotheses testing

### 4.1. Test of the main effect and mediating effect

We also made a multicollinearity diagnosis for all models in the regression analysis. The VIF (Variance Inflation Factor) of all variables was less than 3, far lower than the critical value of 10, indicating that the multicollinearity problem is not serious. The regression analysis results of the main effect and the mediating effect are shown in Table 5.

**Table 5:** Results of the main effect and mediating effect (*n* = 268).

Variable name	Flow experience			Impulse consumption intention				
	Model 1	Model 2	Model 3	Model 4	Model 5	Model 6	Model 7	Model 8
Control variable								
Gender	0.049	0.010	0.022	0.001	−0.053	0.012	−0.058	0.003
Age	−0.055	0.062	−0.029	−0.092	−0.027	−0.081	−0.056	−0.068
Education	−0.085	−0.029	−0.042	0.004	0.020	0.026	0.034	0.044
Independent variable								
Credibility		0.360^***^			0.371^***^		0.210^***^	
Professionalism		0.189^**^			0.386^***^		0.184^**^	
Attractiveness		0.247^***^			0.338^***^		0.113^*^	
Interactivity		0.364^***^			0.269^***^		0.069^*^	
Coexistence presence			0.282^***^			0.318^***^		0.024^*^
Communicational presence			0.221^**^			0.335^***^		0.051^*^
Emotional presence			0.251^***^			0.343^***^		0.234^***^
Mediating variable								
Flow experience							0.466^***^	0.431^***^
*R* ^2^	0.017	0.384	0.433	0.008	0.196	0.204	0.330	0.310
△*R*^2^	0.006	0.367	0.420	−0.003	0.174	0.186	0.309	0.291
*F* value	1.504	23.117^***^	33.247^***^	0.741	9.036^***^	11.170^***^	15.913^***^	16.674^***^

^***^p < 0.001, ^**^p < 0.01, ^*^p < 0.05.

In Table 5, Model 1 and Model 4 only include control variables. Based on Model 1, the characteristics of livestreaming information sources (credibility, professionalism, attractiveness, interaction) are added to form model 2. The credibility of livestreaming information sources has a significant impact on the flow experience (*β* = 0.360, *p* < 0.001). The professionalism of livestreaming information sources has a significant impact on the flow experience (*β* = 0.189, *p* < 0.01). The attraction of livestreaming information sources has a significant impact on the flow experience (*β* = 0.247, *p* < 0.001). The interaction of livestreaming information sources has a significant impact on the flow experience (*β* = 0.364, *p* < 0.001). Therefore, hypothesis 3a, 3b, 3c and 3d are all verified. Based on model 1, the independent variables social presence (coexistence presence, communication presence, emotional presence) are added to form Model 3. The coexistence presence has a significant impact on the flow experience (*β* = 0.282, *p* < 0.001); Communication telepresence has a significant impact on the flow experience (*β* = 0.221, *p* < 0.01); Emotional telepresence has a significant impact on flow experience (*β* = 0.251, *p* < 0.001). Therefore, hypothesis 4a, 4b, and 4c are all supported.

Impulsive consumption intention is the dependent variable from Model 4 to Model 8 in Table 5. Based on Model 4, the characteristics of livestreaming information sources (credibility, professionalism, attractiveness, interactivity) are introduced to form Model 5. It can be seen that the credibility of livestreaming information sources has a significant impact on impulsive consumption intention (*β* = 0.371, *p* < 0.001); The professionalism of livestreaming information sources has a significant impact on impulsive consumption intention (*β* = 0.386, *p* < 0.001); The attraction of live information sources has a significant impact on impulsive consumption intention (*β* = 0.338, *p* < 0.001); The interaction of livestreaming information sources has a significant impact on impulsive consumption intention (*β* = 0.269, *p* < 0.001). Therefore, hypothesis 1a, 1b, 1c, and 1d are supported. Based on Model 4, social presence (coexistence presence, communication presence, emotional presence) is introduced to form Model 6. Coexistence presence has a significant impact on impulsive consumption intention (*β* = 0.318, *p* < 0.001); Communication presence has a significant impact on impulsive consumption intention (*β* = 0.355, *p* < 0.001); Emotional presence has a significant impact on impulsive consumption intention (*β* = 0.343, *p* < 0.001). Therefore, hypothesis 2a, 2b, and 2c are supported. Flow experience is added to Models 5 and 6 to form Models 7 and 8, respectively. Flow experience has a significant impact on impulsive consumption intention (*β* = 0.466, *p* < 0.001; *β* = 0.431, *p* < 0.001), thus Hypothesis 5 is supported.

Based on Model 5, the mediating variable flow experience is added to form Model 7. It can be seen that the characteristics of live information sources have a significant positive impact on impulsive consumption intention, and the influence coefficient and significance of live information source characteristics (credibility, professionalism, attractiveness, interaction) on impulsive consumption intention are reduced (*β* = 0.210, *p* < 0.001; *β* = 0.184, *p* < 0.01; *β* = 0.113, *p* < 0.05; *β* = 0.069, *p* < 0.05), that is, the flow experience plays an mediating role in the relationship between the characteristics of livestreaming information sources and impulsive consumption intention. Thus, hypothesis 6a, 6b, 6c, and 6d are supported. Based on Model 6, the mediating variable flow experience is added to form Model 8. It can be seen that social presence has a significant positive impact on impulsive consumption intention, and the influence coefficient and significance of social presence (coexistence presence, communication presence, emotional presence) on impulsive consumption intention are reduced (*β* = 0.024, *p* < 0.05; *β* = 0.051, *p* < 0.05; *β* = 0.234, *p* < 0.001), which means flow experience plays an mediating role in the relationship between social presence and impulsive consumption intention. Thus, hypothesis 7a, 7b, and 7c are supported.

### 4.2. Test of the moderating effect

In this paper, multiple hierarchical regression analysis is used to test the moderating effect, and the results are shown in Table 6.

**Table 6:** Results of the hierarchical moderated regression analysis (*n* = 268).

Variable name	Flow experience						
	Model 9	Model 10	Model 11	Model 12	Model 13	Model 14	Model 15
Control variable							
Gender	0.049	0.010	0.010	0.026	0.022	0.004	0.021
Age	−0.055	0.062	0.105^*^	0.088	−0.029	0.036	0.033
Education	−0.085	−0.029	−0.053	−0.054	−0.042	−0.064	−0.056
Independent variable							
Credibility		0.360^***^	0.137^*^	0.145^**^			
Professionalism		0.463^***^	0.264^***^	0.277^***^			
Attractiveness		0.480^***^	0.367^***^	0.303^***^			
Interactivity		0.545^***^	0.367^***^	0.384^***^			
Coexistence presence					0.580^***^	0.404^***^	0.416^***^
Communicational presence					0.577^***^	0.413^***^	0.409^***^
Emotional presence					0.524^***^	0.309^***^	0.298^***^
Time pressure			0.361^***^	0.365^***^		0.313^***^	0.315^***^
Moderating							
Credibility × Time pressure				0.165^***^			
Professionalism × Time pressure				0.120^*^			
Attractiveness × Time pressure				0.134^**^			
Interactivity × Time pressure				0.099^*^			
Coexistence presence × Time pressure							0.125^**^
Communicational presence × Time pressure							0.086
Emotional × Time pressure							0.053
*R* ^2^	0.017	0.384	0.471	0.497	0.433	0.494	0.508
Δ*R*^2^	0.006	0.367	0.455	0.473	0.420	0.480	0.489
*F* value	1.504	23.117^***^	28.811^***^	20.959^***^	33.247^***^	36.267^***^	26.560^***^

^***^p < 0.001, ^**^p < 0.01, ^*^p < 0.05.

According to Table 6, from Model 11 and Model 14, time pressure has an impact on cardiac flow experience (*β* = 0.361, *p* < 0.001; *β* = 0.313, *p* < 0.001). Model 12 is formed based on Model 11 by adding the interaction items of live information source characteristics (credibility, professionalism, attractiveness, and interactivity) and time pressure. From the table, it can be seen that the interaction item of live information source credibility and time pressure has a significant positive impact on the flow experience (*β* = 0.165, *p* < 0.001), which means that the increase of time pressure will enhance the positive impact of the credibility of live information sources on the flow experience, so hypothesis 8a is supported; The interaction between the professionalism of live information sources and time pressure on the flow experience (*β* = 0.120, *p* < 0.05) has a significant positive impact, which means that the increase of time pressure will enhance the positive impact of live information source professionalism on cardiac flow experience, thus hypothesis 8b is supported; Interactive item of attraction of live information sources and time pressure on cardiac flow experience (*β* = 0.134, *p* < 0.01) has a significant positive impact, which means that the increase of time pressure will enhance the positive impact of the attraction of live information sources on cardiac flow experience, so hypothesis 8c is supported; Interaction item of live information sources and time pressure on flow experience (*β* = 0.099, *p* < 0.05) has a significant positive impact, which indicates that the increase of time pressure will enhance the positive impact of live information source interaction on cardiac flow experience, the hypothesis 8d hence is supported.

Based on Model 14, the interaction items of social presence (coexistence presence, communication presence, emotional presence) and time pressure are added to form Model 15. From the table, it can be seen that the interaction item of coexistence presence and time pressure has a significant positive effect on cardiac flow experience (*β* = 0.125, *p* < 0.01), which means that the increase of time pressure will enhance the positive effect of coexistence telepresence on cardiac flow experience, so hypothesis 9a is supported; Interactive item of communication telepresence and time pressure on flow experience is not significant (*β* = 0.086, *p* > 0.05), which means that the increase of time pressure will not enhance the positive impact of live information source professionalism on cardiac flow experience, so hypothesis 9b is not supported; The interaction of emotional telepresence and time pressure on flow experience is also not significant (*β* = 0.053, *p* > 0.05), which means that the increase of time pressure will not enhance the positive impact of the attraction of live information sources on the flow experience, so the hypothesis 9c is not supported, too.

## 5. Conclusion and discussion

### 5.1. Result discussion

This paper introduces the S-O-R model into the research of consumers’ impulsive consumption intention in the context of livestreaming e-commerce delivery. It focuses on the impact of livestreaming information source characteristics and social presence on consumers’ consumption behavior and analyzes the antecedents and process mechanisms of consumers’ impulsive consumption intention based on the theories of flow experience and social presence. The main findings and implications are as follows.

#### 5.1.1. The effects of live information sources on consumers’ flow experience and impulsive consumption intention

This first of all, it seems to be a trend for consumers to shop in the livestreaming room. More and more stars and netizens participate in the livestreaming industry to select products for consumers. The inherent traffic foundation and high-quality reputation of stars and netizens make consumers trust the anchor, so it is easier to produce a higher level of flow experience and impulsive consumption intention due to the trust of the anchor.

Secondly, from the perspective of the professionalism of livestreaming information sources, most anchors are familiar with their products in livestreaming room, have professional knowledge reserves and rich use experience, and some anchors have high professionalism. From the perspective of consumers, consumers’ shopping is becoming more and more rational. Compared with false and exaggerated marketing rhetoric, more professional marketing methods will make consumers have a stronger flow experience. The more professional the anchor is, the more abundant the information about products the consumer thinks the anchor has, the lower the perceived risk of selling goods in the live stream room, and the more impulsive the consumer will be.

Third, from the perspective of the attraction of livestreaming information sources, when the anchor has a better appearance, image, or talent, it can increase the popularity of the audience, cause visual impact, be pleasing to the eye, and make the audience feel that watching a live stream is a kind of wonderful entertainment, to produce a streaming experience. When the anchor has an interesting presentation, the audience will also be attracted by it in the process of watching the live stream, resulting in impulsive consumption intention due to love for the anchor.

Finally, the stronger the interaction of the anchor and the richer the forms of interaction, the more conducive it is to enhance consumers’ flow experience and impulsive consumption intention. Under the atmosphere of the livestreaming room, the interaction between the anchor and consumers and between consumers can give consumers a virtual interpersonal interaction experience, enhance consumers’ sense of excitement and pleasure, temporarily forget worldly troubles, and be immersed in livestreaming activities. In addition, when the anchor engages in high-frequency interactions with consumers or between consumers, consumers’ information about product functions and after-sales services will be answered in real time, which is more conducive to consumers’ direct order consumption.

#### 5.1.2. The effects of social presence on consumers’ flow experience and impulsive consumption intention

In the live stream, coexistence, communication, and emotional presence are the three dimensions of social presence in this study. At the same time, these three dimensions are hypothesized and verified on the impact of consumers’ impulsive consumption intention and flow experience respectively, verifying the impact path of the three dimensions of social presence on consumers’ impulsive consumption intention and flow experience. First, the sense of coexistence has a significant positive impact on consumers’ impulsive consumption intention and flow experience. The sense of coexistence refers to the degree to which individuals in the livestreaming room feel the company of others in the livestreaming interaction. If the audience feels that they exist online and have a cross space–time connection with others in the livestreaming room, then the audience watching the live stream is more likely to have impulsive consumption intention and good flow experience. Secondly, the sense of communication presence has a significant positive impact on consumers’ impulsive consumption intention and flow experience. The sense of coexistence presence refers to the degree to which individuals in the livestreaming room feel the smoothness of real-time communication in the live interaction with others, the feeling that viewers socialize on the livestreaming room and can continuously exchange views with others. This experience can improve the flow experience of viewers. If the audience in the livestreaming room can smoothly exchange views with others and their questions can be responded by others in the livestreaming room, the audience will also have impulsive consumption intentions. Finally, emotional presence significantly and positively affects consumers’ impulsive consumption intention and flow experience. Emotional presence refers to the significant degree of emotional relationship between individuals in the livestreaming room and others in the livestreaming interaction. If the livestreaming room brings enthusiasm to the audience, and the live stream of others’ happiness, anger, sadness, and joy can affect the audience’s mood, then the audience is vulnerable to the influence of others, resulting in emotional fluctuations and higher flow experience. An audience is more likely to have impulsive consumption intentions if it is emotionally dependent on a live studio.

Overall, this paper finds that the dimensions of livestreaming information sources and social presence will have a significant impact on consumers’ flow experience and impulsive consumption intention. Therefore, a live stream should focus on improving the livestreaming room. For example, in order to make consumers have a higher flow experience in the livestreaming room, the platform should strictly control the selection process, and let consumers experience value for money every time they consume in the live stream room. Furthermore, maintaining a good interaction between the livestreaming room and consumers will offer consumers a stronger flow experience and prompt an impulsive consumption intention. The livestreaming room should pay attention to the needs of users to the greatest extent. If there are many people online, the backstage staff should also pay attention to users’ questions in time. At the same time, the livestreaming room should carry out more welfare activities; the live studio should vigorously implement blessing bags, preferential activities such as commentary lotteries, and encourage people to join the fan group.

#### 5.1.3. The mediating role of flow experience

When the audience feels a strong flow experience, the loss of sense of time and the increase of positive emotions will immerse consumers into the live stream, make them forget the existence of the external environment, increase the sense of control, and then produce an impulsive consumption intention. This paper empirically finds that the seven characteristics of livestreaming information sources and social presence will stimulate consumers’ flow experience and then produce an impulsive consumption intention. Anchors can focus on livestreaming marketing according to their own conditions and product needs to improve live stream, and the livestreaming room should also be upgraded to create a livestreaming room where the audience can feel the sense of presence. For businesses, they can try to choose stars, online celebrities, or average people with outstanding characteristics as anchors to improve their credibility and attractiveness and improve the sense of coexistence and presence perceived by the audience by transforming the scene layout of the livestreaming room and upgrading the livestreaming process. The anchor not only needs to constantly improve knowledge and professional quality to improve professionalism, but also needs to pay attention to the questions and needs of the audience in the live stream room all the time to improve the interaction with the audience. In addition, the anchor should also form a good emotional communication with the audience and be a “good confidant” to form an emotional telepresence with the audience.

#### 5.1.4. The moderating effect of time pressure

This paper finds that time pressure positively regulates the relationship between the credibility, professionalism, attractiveness, interactivity, and flow experience of live information sources, while time pressure only regulates the sense of coexistence in social presence. Because consumers choose to shop online for the sake of saving time, cost, and convenience, compared with consumers who have enough time to stay in the livestreaming room for careful selection, in the limited shopping time, these consumers under time pressure are more likely to have a stronger flow experience from the credibility, professionalism and attractiveness of the host. When encountering the promotional activities that need to race against time, such as the second kill in the livestreaming room and the rush purchase, the high time pressure brought by the active interaction between consumers and the anchor when participating in the activities will further promote a strong flow experience for consumers. When consumers feel that there are online and share time and space connections with others at the same time, the doubling of time pressure will lead to changes in consumers’ feelings, which will affect the flow experience. When consumers communicate with others in the livestreaming room and feel the changes in their emotions, the speed of time does not affect their flow experience.

Based on the above analysis, this paper suggests that brands settling in e-commerce and livestreaming platforms should actively participate in livestreaming promotion activities of various festivals. In the context of high time pressure and complex discount rules such as festival promotion, relying on the characteristics of the anchor and creating strong coexistence in on-site perception, they should try their best to display commodity information in the livestreaming room in a real and comprehensive way to improve the flow experience of users, and then improve the decision-making quality of consumers watching the live stream. In addition to the promotional activities of the Shopping Festival live stream, the daily live stream should also have high time pressure activities such as second kill and rush purchase. These activities should be held many times in a live stream as much as possible. Finally, all brands should make all anchors reach a unified consensus, that is, when the anchor explains the goods in the livestreaming room, they should do so in a manner that does not require the consumer to spend too much time to understand the goods and help consumers who do not have enough time to watch the live stream to save time.

### 5.2. Theoretical contributions

The contribution of this study is twofold: first, this study analyzes the state and response of consumers when watching the live stream from the aspects of information source characteristics, social presence, time pressure, and flow experience, and confirms that flow experience has a significant positive impact on consumers’ impulsive consumption wishes. It reveals the mediating role of flow experience between the characteristics of livestreaming information sources and social presence on consumers’ impulsive consumption intention and demonstrates the moderating effect of time pressure in the process of the impact of livestreaming information source characteristics and social presence on flow experience. Since the research of livestreaming experience in the e-commerce field is still rare, this paper deeply analyzes how the characteristics of livestreaming information sources and social presence affect the internal state and feelings of consumers based on streaming theory and explores the mechanism between the characteristics of livestreaming information sources and social presence on consumers’ impulsive consumption intention. It provides a new theoretical perspective and research findings for further understanding and studying the impact of livestreaming information source characteristics and social presence on consumption behavior.

Second, this paper also enriches the research literature on the characteristics of information sources and social presence in the field of livestreaming e-commerce. This study points out that anchors play a vital role in the transaction volume of livestreaming rooms and consumers’ purchase decisions, as a key figure connecting enterprises, livestreaming platforms, and consumers. Based on the theory of information source characteristics, anchors are defined as livestreaming information sources, and this study analyses the influence mechanism of their characteristics on consumers’ impulsive consumption intention. Secondly, based on the strong social presence of livestreaming marketing, taking social presence as the independent variable, the social presence is divided into three dimensions to study its mechanism of action on impulsive consumption intention.

Finally, in the relevant literature on e-commerce live stream, time pressure is rarely included in the influencing factors of consumers’ impulsive consumption. This study expanded the existing literature by investigating the influence mechanism of time pressure between the characteristics of live stream information source and flow experience, social presence, and flow experience. Because consumer buying behavior is the result of multiple factors including product participation and time pressure in a real live stream scenario, it is important to consider these factors for a more detailed analysis. The current research found that time pressure has some influence mechanism between the two groups of variables, but the influencing factors under each group of variables are different, which provides a research perspective and theoretical basis for the follow-up study on impulsive consumption behavior of time pressure in e-commerce live stream.

### 5.3. Managerial implications

The research findings of this paper have three practical implications:

Firstly, this study not only has certain significance for the theoretical literature of impulsive consumption of e-commerce live stream, but also has certain practical significance for the enterprise management of e-commerce live stream.

#### 5.3.1. Strengthen the professionalism and credibility of the anchor in the livestream room

For enterprises, seize the opportunity of e-commerce development, increase investment in e-commerce live stream, tap professional and credible anchors and reach cooperative relations with anchors with these characteristics to increase product sales; In addition, enterprises should also cultivate their own anchors so that they not only have good anchor characteristics, but also know more about their products. Therefore, enterprises need to cultivate the professional quality of anchors, conduct regular training and assessment, improve their understanding of the related fields of their products, and position themselves according to their own personality characteristics and images, find a suitable development path, enhance the influence of anchors, and maximize the effectiveness of e-commerce anchor marketing.

#### 5.3.2. Enrich the attraction of live stream

Enterprises should let consumers feel the self-efficacy brought by using live stream, which may make consumers use live stream software to purchase products recommended by anchors more continuously therefore, the live stream content should be more life-oriented and personalized. The e-commerce department of the enterprise should formulate attractive marketing methods. These means can be to remind the audience to pay attention to the anchor’s task of joining the “fan group” and receiving the red envelope of fans, to improve the stickiness and use experience of fans, increase the use efficiency of viewers, and improve their emotional arousal of watching live stream. It can also make the content of the live stream room more unique, so that the anchor can display talents, skills, and special resources richly, so that consumers can have feelings of love, admiration and worship in self-comparison, and traffic can drive the sales of products and services.

#### 5.3.3. Deepen the interaction with consumers

Enterprises should let anchors make full use of the sociality of the live stream platform and pay more attention to the communication with users. Combine the platform used to determine the appropriate communication style pay attention to the real-time interactive feedback of consumers, improve the service quality of anchors, and improve the use efficiency of users let consumers realize that the sense of existence compensation cannot directly perceive the shortcomings of goods through the senses, enhance the activity of the live stream platform and the anchor interface, and attract more consumers to join.

#### 5.3.4. Create a fascinating sense of social presence

Enterprises should take information technology as an interactive medium and transmission bridge with consumers, develop convenient and easy-to-control communication channels, clear layout and concise links, and improve the social presence of the live stream platform, so that consumers can feel immersive Live e-commerce should set off the scarcity atmosphere of products in the live stream room, so that consumers can perceive the usefulness, effectiveness and fun of products and stimulate consumers’ impulse to buy through limited marketing strategies. The use scenarios of live stream products should be as rich as possible. The anchor should fully display the multi-purpose of the product so that consumers can experience the product empathetically. At the same time, the live stream platform should also be designed to embed experiential elements to provide an easy-to-operate personalized and interesting platform interface from the perspective of consumers to deepen consumers’ pleasure and immersion.

#### 5.3.5. Let consumers feel the “time pressure”

Nowadays, low prices have become the main selling point of e-commerce live stream. Live prices, that is, low prices, have been deeply rooted in the hearts of the people enterprises use marketing methods such as limited time spike for 7 days, no reason to return and exchange goods, transportation insurance fees, etc., so that consumers can make decisions in a short time. If consumers feel that they will lose their own interests if they do not place orders immediately, then consumers will make impulsive consumption. Therefore, merchants can carry out profit-making marketing under the condition of controllable cost, train anchors to promote in a limited time in the live stream room and have a sense of urgency. In the live stream, activities such as issuing irregular coupons and snapping up in a limited time stimulate the time pressure felt by consumers.

Secondly, for consumers, because they can more intuitively understand their reactions when watching the live stream, they can better control their impulsive consumption intention by understanding the mechanism and influencing factors of impulsive consumption intention. At the same time, consumers should also constantly improve their ability to distinguish and select, keep a clear head, and refuse to just follow the crowd and consume blindly.

Finally, the government and the livestreaming platform regulators should strictly control the content, words and deeds of the anchor’s live stream, and establish and improve the binding policies on live stream. Although the rise of livestreaming e-commerce occurred recently, with the rapid development of the webcast, false publicity, trust crisis, inadequate after-sales service and other problems appear frequently. Therefore, relevant institutions should standardize the livestreaming order, establish a trust mechanism, raise the access threshold of the live stream room, formulate relevant industry constraints and norms, establish a punishment mechanism for publishers and propagandists (including anchors) of false information, strictly protect the legitimate rights and interests of consumers, and create a healthy and benign environment of livestreaming e-commerce for the public.

### 5.4. Limitations and further research

This paper also has some limitations. First, the content of the questionnaire was filled in by the participants based on their recent live viewing experiences, not immediately after watching the live video. Therefore, there is a certain degree of backtracking deviation from the choice in the real scenario. Future research could use an experiment method to conduct a questionnaire, such as providing subjects with a live situation description or live playback video to help them express their true feelings when watching e-commerce live video, and then fill in the questionnaire to better predict consumers’ feelings when watching live videos and their purchase decisions. Secondly, the formation mechanism of consumers’ impulsive consumption intention is not a simple process. In addition to flow experience and time pressure, there will also be other factors that affect the characteristics of livestreaming information sources, social existence, and the relationship between consumers’ impulsive consumption intention. Finally, whether there are unexplored areas in the dimension division of livestreaming information source characteristics and social presence is also worth further exploration in future research combined with the situational particularity of livestreaming e-commerce.

## Data availability statement

The raw data supporting the conclusions of this article will be made available by the authors, without undue reservation.

## Author contributions

W-wD contributed to the conception and design of the study and revised the first draft. Y-qW conducted statistical analysis and wrote the first draft of the manuscript. JQ contributed to the analysis and interpretation of the data, and further revised the contents of the manuscript. All authors contributed to the article and approved the submitted version.

## Funding

This study was supported by the National Natural Science Foundation of China (No. 71602119; PI: W-wD; Grant Title: The relational inertia dilemma of trust overdevelopment in marketing channels and its governance strategies).

## Conflict of interest

The authors declare that the research was conducted in the absence of any commercial or financial relationships that could be construed as a potential conflict of interest.

## Publisher’s note

All claims expressed in this article are solely those of the authors and do not necessarily represent those of their affiliated organizations, or those of the publisher, the editors and the reviewers. Any product that may be evaluated in this article, or claim that may be made by its manufacturer, is not guaranteed or endorsed by the publisher.
